# Mutations in SARS-CoV-2 ORF8 Altered the Bonding Network With Interferon Regulatory Factor 3 to Evade Host Immune System

**DOI:** 10.3389/fmicb.2021.703145

**Published:** 2021-07-16

**Authors:** Farooq Rashid, Muhammad Suleman, Abdullah Shah, Emmanuel Enoch Dzakah, Haiying Wang, Shuyi Chen, Shixing Tang

**Affiliations:** ^1^Dermatology Hospital, Southern Medical University, Guangzhou, China; ^2^Guangdong Provincial Key Laboratory of Tropical Disease Research, School of Public Health, Southern Medical University, Guangzhou, China; ^3^Center for Biotechnology and Microbiology, University of Swat, Mingora, Pakistan; ^4^Department of Biotechnology, Shaheed Benazir Bhutto University, Sheringal, Pakistan; ^5^Department of Molecular Biology and Biotechnology, School of Biological Sciences, College of Agriculture and Natural Sciences, University of Cape Coast, Cape Coast, Ghana; ^6^Wenzhou Institute, University of Chinese Academy of Sciences, Wenzhou, China

**Keywords:** SARS-CoV-2, ORF8 mutants, IRF3, protein–protein docking, MD simulation

## Abstract

Severe acute respiratory syndrome coronavirus 2 (SARS-CoV-2) has been continuously mutating since its first emergence in early 2020. These alterations have led this virus to gain significant difference in infectivity, pathogenicity, and host immune evasion. We previously found that the open-reading frame 8 (ORF8) of SARS-CoV-2 can inhibit interferon production by decreasing the nuclear translocation of interferon regulatory factor 3 (IRF3). Since several mutations in ORF8 have been observed, therefore, in the present study, we adapted structural and biophysical analysis approaches to explore the impact of various mutations of ORF8, such as S24L, L84S, V62L, and W45L, the recently circulating mutant in Pakistan, on its ability to bind IRF3 and to evade the host immune system. We found that mutations in ORF8 could affect the binding efficiency with IRF3 based on molecular docking analysis, which was further supported by molecular dynamics simulations. Among all the reported mutations, W45L was found to bind most stringently to IRF3. Our analysis revealed that mutations in ORF8 may help the virus evade the immune system by changing its binding affinity with IRF3.

## Introduction

Severe acute respiratory syndrome coronavirus 2 (SARS-CoV-2) causes the current pandemic of coronavirus disease 2019 (COVID-19) and is phylogenetically related to SARS-CoV, the cause of 2002–2003 severe acute respiratory syndrome and other bat-related SARS-CoVs (Ceraolo and Giorgi, 2020). It consists of 12 open-reading frames (ORFs), which encode 4 structural and 22 non-structural proteins (Wang M. Y. et al., 2020). The structural proteins are nucleocapsid (N), membrane (M), envelop (E), and spike (S). The non-structural proteins (nsp) contain 1–16 nsp and are encoded by ORF1ab and six accessory proteins, i.e., ORF3a, ORF6, ORF7a, ORF7b, ORF8, and ORF9 (Wu et al., 2021).

In general, viral infection is hindered by the activation of the type 1 interferon (IFN) pathway. The viral pathogen-associated molecular patterns are recognized by the host pattern recognition receptors. As a result, interferon regulatory factor 3 (IRF3) is activated (Chen et al., 2014). IRF3 resides in the cytoplasm in inactive form. However, when it is activated by pathogen infection, IRF3 is phosphorylated and translocates to the nucleus (Sharma et al., 2003), where it binds to the conserved sequences known as IFN stimulated response elements to induce the transcription of type I IFN genes (Kawai and Akira, 2007). Finally, interferon-stimulated genes are activated, which are important in controlling early infections (Ivashkiv and Donlin, 2014).

Viruses, including coronaviruses, have developed strategies to suppress IFN production by targeting different aspects of IFN signaling for successful infection (Lim et al., 2016). Viral proteins help coronaviruses to suppress the host innate immune system by binding to IRF3, which, in turn, inhibits the production of IFNß (Wathelet et al., 2007; Huang et al., 2011; Zhong et al., 2012; Liu et al., 2014; Lim et al., 2016). Moreover, viruses especially RNA viruses, including SARS-CoV and SARS-CoV-2, undergo rapid mutations, which benefit virus dissemination and infection (Lu et al., 2020).

Among all accessory proteins of SARS-CoV-2, ORF8 shows intriguing characteristics and is more prone to mutating. This protein has 366 nucleotides and 121 amino acids (Pereira, 2020). Recently, several studies showed that ORF8 could downregulate the major histocompatibility complex class I (MHC-I; Zhang et al., 2021) and the type I IFN signaling pathway to evade the host immune system by decreasing the nuclear translocation of IRF3 (Li et al., 2020; Rashid et al., 2021; Flower et al., 2021). Several mutations in SARS-CoV-2 ORF8 protein have been identified, i.e., ORF8 L (Leucine) and ORF8 S (Serine) at amino acid 84 (Ceraolo and Giorgi, 2020; Tang et al., 2020); ORF8 V (Valine) and ORF8 L at amino acid 62; and ORF8 S and ORF8 L at amino acid 24 (Laha et al., 2020). Moreover, it has been found that V62L mutation was accompanied with L84S mutation (Laha et al., 2020). Recently, another ORF8 mutation was also identified, i.e., ORF8 W (Tryptophan) and ORF8 L at amino acid 45, and is currently circulating in the virus isolates (“Indian variant”) in Pakistan (The Genbank accession number MW447642.1).

Since ORF8 protein affects several host cellular processes and has evolved strategies that help it to evade the host immune system (Rashid et al., 2021), therefore, it was important to investigate whether a wild type (WT) or the different mutations in ORF8 could affect the interaction with IRF3 and antagonize IFNß. In the current study, we adapted comparative binding and biophysical approaches to assess the role of patient-derived ORF8 mutations in host immune evasion through its binding with IRF3. We found that mutations in ORF8, in particular W45L, increase the binding with IRF3, suggesting the important role of SARS-CoV-2 ORF8 in regulating innate immune response upon virus infection.

## Materials and Methods

### Data Retrieval and Variants Modeling

The crystal structure of ORF8 (PDB ID: 7JTL) was retrieved from UniProt (Magrane and UniProt Consortium, 2011). The Chimera software (Goddard et al., 2005) was used to simulate the structural model of SARS-CoV-2 ORF8 WT with other mutants (S24L, W45L, V62L, and L84S) based on the structure of ORF8 WT. The crystal structure of IRF3 was retrieved from UniProt (Magrane and UniProt Consortium, 2011).

### Protein–Protein Docking

A high ambiguity-driven protein–protein docking (HADDOCK) algorithm was used for protein–protein (ORF8 WT/mutants-IRF3) docking to check the binding efficiency of the ORF8 protein with the human IRF3 protein. The Guru Interface was used to visualize the docking interface, i.e., about 500 features for protein–protein, protein–DNA, and protein–RNA docking. The Guru interface is the best interface operated by the HADDOCK server (Xue et al., 2016).

### Molecular Dynamics Simulation

Amber20 was used to perform the dynamic behavior analysis of ORF8 WT and ORF8 with mutations of S24L, W45L, V62L, and L84S through MD simulation (Salomon-Ferrer et al., 2013) that uses FF14SB force field. We used the TIP3P water box to perform the system solvation, and the counter ions were added to neutralize the system (Price and Brooks, 2004). For bad clashes removal in the system, an energy minimization protocol was used. The steepest descent algorithm was used for 6,000 cycles (Meza, 2010), and the conjugate gradient algorithms were used for 3,000 cycles (Watowich et al., 1988). The system was equilibrated at 1 atm constant pressure with weak restraint after 300 K heating. Afterward, the molecular dynamics simulation was run for 100 ns. The particle mesh Ewald algorithm (Salomon-Ferrer et al., 2013) was used to treat the long-range electrostatics integrations with a 10.0 Å cutoff distance. However, to treat covalent bonds, the SHAKE algorithm was used (Krautler et al., 2001). The CUDA and trajectories were analyzed by the Amber20 CPPTRAJ package, while the molecular dynamics simulations were carried out on PMEMD (Roe and Cheatham, 2013).

### Binding Free Energy Calculations

The MMGBSA approach was used to analyze the actual binding energy of ORF8 WT and ORF8 mutants with IRF3. The MMGBSA is the most suitable approach used by different studies for estimating various binding complexes, such as protein–protein, protein–DNA, and protein–RNA (Khan et al., 2018, 2019, 2021; Ali et al., 2019). The total free energy, GB, SA, electrostatic, and vdW of the ORF8 WT and ORF8 mutants were calculated by using the script MMGBSA.py (Hou et al., 2011).

The following equation was used for free energy calculations:

ΔG⁢(bind)=ΔG⁢(complex)-[ΔG⁢(receptor)+ΔG⁢(ligand)]

Each component of the total free energy was estimated by the following equation:

G=Gbond+Gele+GvdW+Gpol+Gnpol

In the above equation, Gbond stands for bond, Gele for electrostatic, and GvdW for van der Waals interactions. Gpol is the polar solvated free energy, while Gnpol is the non-polar solvated free energy. Generalized born (GB) implicit solvent method with the solvent-accessible surface area SASA term was used to calculate Gpol and Gnpol.

## Results

### ORF8 Mutant Modeling and Superimposition on ORF8 WT

**FIGURE 1:**
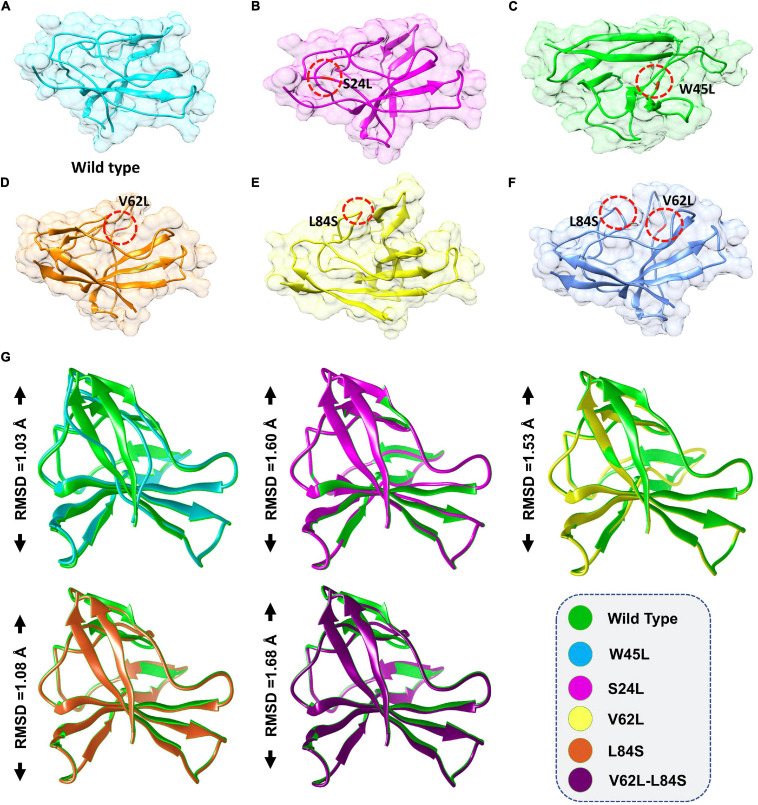
ORF8 mutants modeling and the superimposition of ORF8 WT with ORF8 mutants. **(A)** ORF8 WT, **(B)** S24L, **(C)** W45L, **(D)** V62L, **(E)** L84S, **(F)** V62L, and L84S double mutant **(G)**. Superimposed structure of ORF8 WT (green) with S24L (light magenta), W45L (cyan), V62L (yellow), L84S (orange), and V62L, L84S (dark magenta). The RMSD values of each superimposition were shown.

To investigate whether ORF8 WT or ORF8 mutants could affect their interaction with IRF3, we generated S24L, W45L, V62L, L84S, and V62L-L84S double mutants by using Chimera (Figures 1A–F). The generated mutants were superimposed on the ORF8 WT protein, and the RMSD values were recorded (Figure 1G). The ORF8 WT sequence used in this study has an accession number of MN908947.3. For each superimposed structure, the RMSD differences were substantial with more than 1.0 Å. The mutations in ORF8 altered the protein conformations and the secondary structural elements. Hence, it is essential to understand how these changes may affect the binding of ORF8 and IRF3. Herein, structural approaches, i.e., protein–protein docking and biophysical simulations, were used to precisely estimate the impact of these variations.

### ORF8 WT-IRF3 and ORF8 Mutants-IRF3 Docking

Given the role of the ORF8 protein in host immune system evasion and IRF3 in regulating IFNß production, binding analysis for ORF8 WT or ORF8 mutants with IRF3 was performed. Most biological processes in the cells are regulated by the interaction of different complexes they target for further downstream effects (Ray, 2014). Structural determinants and binding energies determination of these interactions are pivotal steps toward a deeper understanding and regulations of these processes. Importantly, binding affinity, which is the key element for regulating molecular interactions, developing novel therapeutics or predicting the effect of variations on protein interfaces determines whether the complex formation occurs under specific circumstances (Smith and Sternberg, 2002). HADDOCK was used to perform the protein–protein docking of IRF3 with the ORF8 WT and ORF8 mutants including S24L, W45L, V62L, L84S, and V62L and L84S double mutants to unwind the structural mechanisms behind the higher infectivity of different variants of SARS-CoV-2.

**FIGURE 2:**
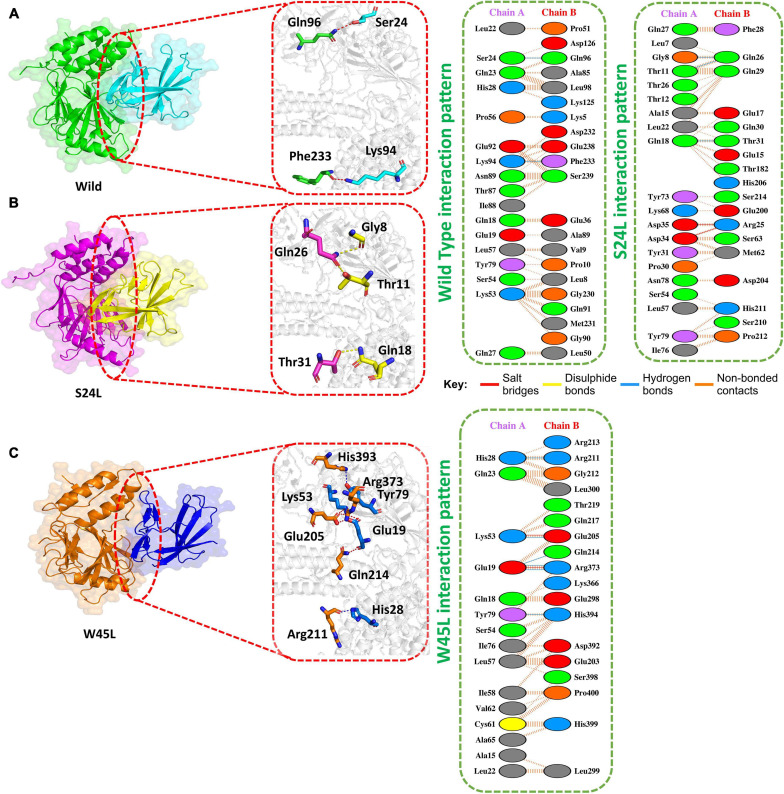
Docking of ORF8 WT, S24L, and W45L mutant complexes. **(A)** Key hydrogen bonding interactions of the ORF8 WT complex with the IRF3 binding interface along with stick representation of the ORF8 WT complex with IRF3 (left). 2D interaction representing salt bridges, hydrogen, and non-bonded interactions (right). **(B)** Key hydrogen bonding interactions of S24L mutant with the IRF3 binding interface along with stick representation (left). 2D interactions representing salt bridges, hydrogen, and non-bonded interactions (right). **(C)** Key hydrogen bonding interactions of the W45L complex with the IRF3 binding interface along with stick representation of the ORF8 WT complex with IRF3 (left). 2D interaction representing salt bridges, hydrogen, and non-bonded interactions (right).

Previous work with ORF8 WT revealed its role in IFNß antagonism (Li et al., 2020; Rashid et al., 2021). HADDOCK predicted the docking score of −293.65 kcal/mol for the IRF3–ORF8 WT complex. Interaction analysis explored through the PDB sum delineated that 38 residues form the interface, among which 21 residues were contributed by the IRF3, while 17 residues were contributed by ORF8 WT. The interaction analysis explored that both structures formed 2 hydrogen bonds and 150 non-bonded interactions. These hydrogen bonds formed by the IRF3–ORF8 WT include Gln96-Ser24 and Phe233-Lys94 (Figure 2A).

The second most frequent mutation in SARS-CoV-2 ORF8 is S24L and accounts for 94.2% of the mutant sequences recorded in the United States of America (Wang R. et al., 2020). The HADDOCK docking score for S24L (IRF3-ORF8) is −321.26 kcal/mol. The substituted residue increases the binding of ORF8 with IRF3. Three hydrogen bonds, two salt bridges and 131 non-bonded contacts were reported (Figure 2B). Gln26-Gly8, Gln26-Thr11, and Thr31-Gln1, which are key residues, formed the hydrogen bonds. Asp35-Arg25 and Asp34-Arg25 residues were involved in the formation of salt bridges. The S24L mutation was found to enhance the function of the ORF8 protein (Wang R. et al., 2020); therefore, it could be speculated that S24L will antagonize IFNß more strongly and hinder the eradication of SARS-CoV-2. However, further experiments are needed to determine whether S24L binds to IRF3 more efficiently compared to ORF8 WT and antagonize IFNß more efficiently.

The mutation W45L in ORF8 was first reported in Saudi Arabia to cause more severe disease and may affect the function of this protein (Hassan et al., 2021). Therefore, it was speculated that W45L mutation may increase its binding to IRF3. The HADDOCK docking score for W45L (IRF3-ORF8) was −351.49 kcal/mol. The molecular interaction of this complex revealed an interaction interface with two salt bridges, while six hydrogen bonds and 134 non-bonded contacts formed by the substituted residue W45L. This mutation indeed increased the binding with IRF3. The salt bridges were formed by the key residues Lys53-Glu205 and Glu19-Arg37. Among the hydrogen bonds, His28-Arg211, Lys53-Glu205, Glu19-Gln214, Glu19-Arg37, and Tyr79-His394 residues were involved (Figure 2C). The docking results indicated a strong interaction of ORF8 protein with W45L mutation, suggesting that this mutation may further increase the function of the ORF8 protein in the evasion of the host immune system.

**FIGURE 3:**
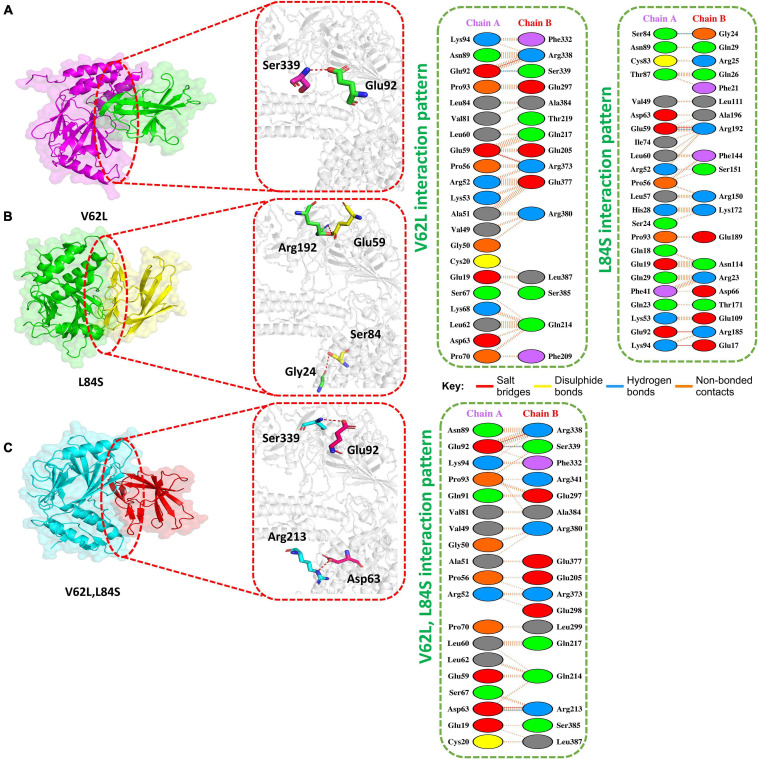
Docking V62L, L84S, and V62L and L84S double-mutant complexes. **(A)** Key hydrogen bonding interactions of the V62L mutant with the IRF3 binding interface along with stick representation (left). 2D interactions representing salt bridges, hydrogen, and non-bonded interactions (right). **(B)** Key hydrogen bonding interactions of the L84S complex with the IRF3 binding interface along with stick representation of the ORF8 WT complex with IRF3 (left). 2D interaction representing salt bridges, hydrogen, and non-bonded interactions (right). **(C)** Key hydrogen bonding interactions of double mutants V62L and L84S with the IRF3 binding interface along with stick representation (left). 2D interactions representing salt bridges, hydrogen, and non-bonded interactions (right).

The HADDOCK docking score for L84S (IRF3-L84S) was reported to be −301.28 kcal/mol. The molecular interaction of this complex revealed an interaction interface with three salt bridges, two hydrogen bonds, and 157 non-bonded contacts formed by L84S. This mutation increased the binding of the IRF3–mutant protein complex as compared to the ORF8 WT complex. However, there was no significant difference in the nuclear translocation of IRF3 after overexpressing ORF8 WT or ORF8 L84S in HEK-293T cells (Rashid et al., 2021). The salt bridges were formed by the key residues Glu59-Arg192, Glu92-Arg185, and Lys94-Glu17. Gly24-Ser84 and Arg192-Glu59 residues were involved in the hydrogen bond formation (Figure 3A).

The V62L mutation was predicted to be neutral (Hassan et al., 2021). Therefore, it was intriguing to assume that this mutation will not affect the function of ORF8. The predicted score of HADDOCK for V62L (IRF3-V62L) was −345.84 kcal/mol. The PDB sum analysis of the complex delineated that 44 residues form the interface. Among these residues, 23 were contributed by IRF3 and 21 residues by ORF8. The interaction analysis found that the two structures form two salt bridges, one hydrogen bond, and 130 non-bonded interactions. Ser339-Glu92 was responsible for forming the hydrogen bonds by IRF3-V62L-ORF8, whereas the salt bridges included Glu92-Arg338 and Glu59-Arg373 residues (Figure 3B).

It was reported that V62L mutation was accompanied with L84S mutation in ORF8 (Laha et al., 2020). Therefore, we want to investigate if a double-mutant ORF8 could increase the immune evasion capability of ORF8 by strongly binding to IRF3. The predicted score of HADDOCK for the double-mutant V62L and L84S (IRF3-V62L-L84S double mutants) complex was −325.79 kcal/mol, which was comparable to that for the ORF8 WT complex. This observation revealed that ORF8 containing the double mutations of V62L and L84S may not affect the function of the ORF8 protein. The PDB sum analysis of the complex revealed that 36 residues form the interface. Among these residues, 17 were contributed by IRF3 and 19 by ORF8. The interaction analysis indicated that the two structures formed two salt bridges, two hydrogen bonds, and 100 non-bonded interactions. The hydrogen bonds formed by the IRF3-V62L, L84S ORF8 included Arg213-Asp63 and Ser339-Glu92, while the salt-bridge included Glu92-Arg338 and Asp63-Arg213 residues (Figure 3C).

### Structural Dynamic Features of ORF8 WT and Mutant Complexes

**FIGURE 4:**
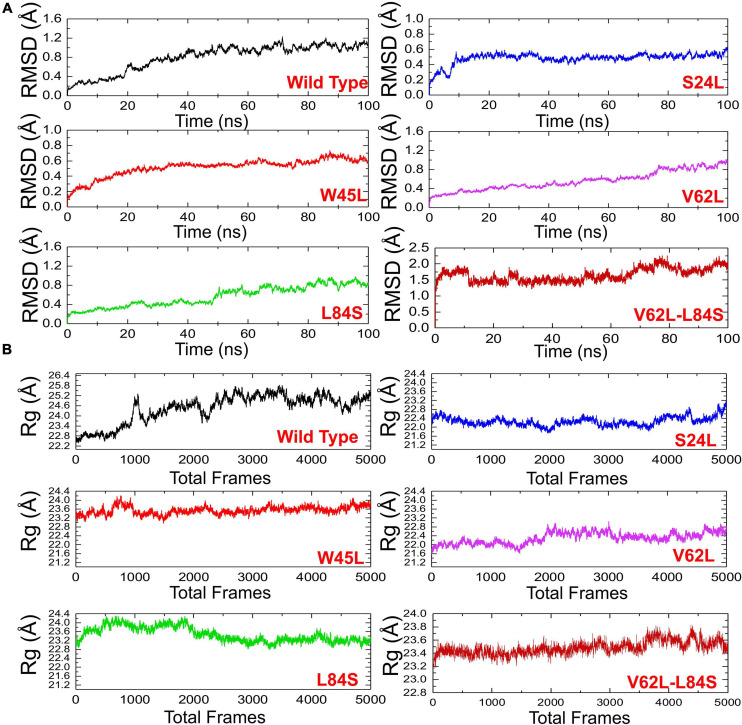
The RMSDs and *Rgs* of all complexes. **(A)** The RMSD of ORF8 WT was shown in black, while other mutants in different colors. RMSDs, root mean square deviations. **(B)** *Rg*(s) of ORF8 WT was shown in black, while the other mutants were given in different colors. Rg, Radius of gyration.

The structural dynamic characterization of the WT and mutant complexes was performed to understand the thermodynamics stability, structural compactness, and residual flexibility. We also calculated the total number of hydrogen bonds to understand the impact of these natural substitutions on the binding of ORF8 to IRF3. To estimate the stability of each complex, the root mean square deviation (RMSD) of each complex with respect to time was calculated (Figure 4A). The overall results showed that all the complexes exhibit rigid structures except the double mutant (V62L-L84S). In the case of the WT, the structure did not attain the equilibrium, and the RMSD continues to increase over the simulation time. During the 100-ns simulation, the structure faces substantial convergences, and significant deviation from the mean position was largely experienced. The average RMSD was observed to be 0.6 Å. On the other hand, the S24L mutant structure abruptly converged after reaching 10 ns, and the RMSD increased from 0.2 to 0.4 Å. Afterward, the structure did not face any convergences and remained uniform over the 100-ns simulation time. Comparatively, the S24L structure remained more stable than the WT. Similarly, the W45L complex followed a similar pattern as the WT, and the RMSD observed was about 0.6 Å; however, the system remained more stable, though the RMSD remained higher and increased continuously. No significant convergence was observed during the simulation. Similarly, the V62L also exhibits a rigid structure, and the RMSD continues to increase over time. The average RMSD remained 0.4 Å. The behavior of L84S exhibits some convergence from the mean position during the first 50 ns. The RMSD then abruptly converged and increased. The double mutant lost the rigidity, and the RMSD remained the highest. The structure also faced significant convergences at different intervals. The average RMSD remained 1.5 Å. The observed simulation pattern is an indication of rigid binding between ORF8 and IRF3. The fixed amino acid substitution facilitated the stable evolution of viral protein, while its binding affinity with the host protein is stronger.

The radius of gyration (*Rg*) was calculated to evaluate the compactness of the protein structure during the simulation (Figure 4B). In the case of the WT, the structure remained open, and the *Rg* value continuously increased during the simulation. The average *Rg* value was 23.40 Å. Unlike the ORF8 WT, the mutant complexes remained more compact. The average *Rg* values for each mutant S24L, W45L, V62L, L84S, and V62L-L84S were 22.40, 23.20, 22.00, 23.20, and 23.40 Å, respectively. These results indicate that the mutant complexes efficiently bound the ORF8 and IRF3 to ensure the suppression of IRF3 and thus eventually lead to the immune evasion. The binding and unbinding of one or both ends of the ORF8 cause the *Rg* value to fluctuate during the simulation of all the structures.

**FIGURE 5:**
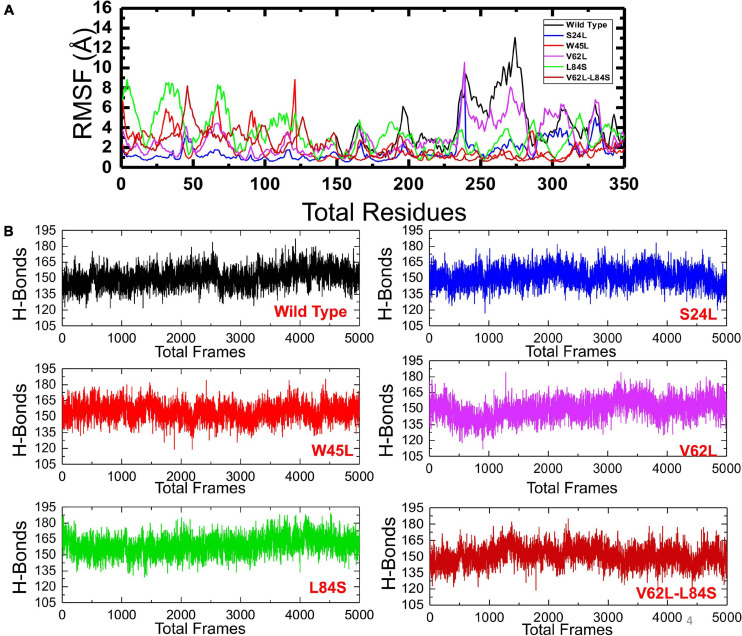
The RMSFs and H-bonding graphs of all complexes. **(A)** The RMSFs of the ORF8 WT were shown in black, while the other mutants were given in different colors. RMSFs, root mean square fluctuation. **(B)** The H-bonding graph of the ORF8 WT was shown in black, while the other mutants were given in different colors.

To understand the dynamics and function relationship as a consequence of the evolutionary divergence of protein motions, the RMSF value of backbone C-alpha was calculated and compared. A large RMSF value is an indication of a flexible region with movements, whereas a low RMSF value suggests a rigid region and minimal movements during the simulation. It can be seen that the region between 1 and 125 fluctuated in all the complexes, while the region between 126 and 240 exhibits minimal fluctuation (Figure 5A). Afterward, the region between 241 and 350 possesses substantial fluctuation particularly in the case of ORF8 WT. According to these results, it can be inferred that the binding of mutant ORF8 with IRF3 stabilized the binding, whereas the residual fluctuation is minimized. The overall results suggest the possible evolutionary changes in the mutants for better binding, which leads to enhanced infectivity and host immunity evasion.

To further understand the impact of these mutations on the change of the total hydrogen bonding and binding affinity, we performed hydrogen bonding analysis and free energy calculations using the MM-GBSA approach. The total number of hydrogen bonds during the 100-ns simulation for each mutant remained variable (Figure 5B). It was observed that the number of H-bonds in the ORF8 WT complex is 152 and is less than in the mutant complexes in which the average H-bonds are 157, 161, 159, 165, and 154 for the mutants S24L, W45L, V62L, L84S, and V62L-L84S, respectively. These results validated the previous docking results and further proved that protein conformational evolution alters the binding of ORF8 to IRF3. For the estimation of real-time binding energy, the MM-GBSA approach was adapted by considering 5,000 snapshots from the simulation trajectories of each complex. The binding energies for ORF8 WT, mutant S24L, W45L, V62L, L84S, and V62L-L84S are −28.47, −45.18, −55.77, −55.66, −35.61, and −41.47 kcal/mol, respectively (Table 1), and are dominated by the van der Waals forces, while the electrostatic energies did not significantly influence the binding.

**TABLE 1:** MM/GBSA binding free energies of the ORF8 WT and ORF mutants.

Complex	VDWAALS	EEL	EGB	ESURF	Total
Wild type	−63.70	3.533	40.76	−9.064	−28.47
S24L	−97.82	81.35	−13.24	−15.47	−45.18
W45L	−111.49	85.06	−13.83	−15.51	−55.77
V62L	−108.76	90.12	−19.21	−17.81	−55.66
L84S	−77.89	84.10	−31.01	−10.80	−35.61
V62L-L84S	−91.47	76.34	−11.11	−15.23	−41.47

*All energy values are presented in kcal/mol.*

## Discussion

Severe acute respiratory syndrome coronavirus 2 has undergone several mutations since it first emerged. With the passage of mutations, the virus became more infectious and got more strength in infectivity (Chen et al., 2020). Theses mutations have a direct correlation to clinical outcomes, are responsible for the spread of virus, and may cause a more severe disease. So far, these mutations occurred in structural, non-structural, and accessory proteins of SARS-CoV-2 (Nagy et al., 2021). The accessory protein, i.e., the ORF8 protein sequence of SARS-CoV-2, has the least homology with that of SARS-CoV (Zhang et al., 2021). In the current study, we adapted structural and biophysical analysis approaches to explore the impact of various mutations of ORF8, such as S24L, W45L, V62L, and L84S, on its ability to bind IRF3 and to evade the host immune system.

The ORF8 protein is one of the fast evolving viral proteins in beta coronaviruses (Flower et al., 2021). Some of the functions attributed to ORF8 include inhibition of IFN-1 signaling and downregulation of MHC-I in cells (Li et al., 2020; Rashid et al., 2021; Zhang et al., 2020b, 2021). Moreover, ORF8 can also stimulate the immune system to produce strong humoral and cellular immune responses upon virus infection, which are the biomarkers for SARS-CoV-2 infection (Flower et al., 2021). These observations suggested that the sequence variations in ORF8 may be pivotal for the roles of different ORF8 mutants in evading the host immune system.

Severe acute respiratory syndrome coronavirus 2 first emerged in Wuhan City of China in early 2020. Since then, it acquired many genetic variations. The genetic alterations have changed its pathogenicity, infectivity, and epidemic (Khan et al., 2021). The ORF8 protein is the most rapidly mutating protein (Flower et al., 2021). The different mutations reported in ORF8 are S24L, W45L, V62L, L84S, V62L, and the V62L/L84S double mutant. These mutations in ORF8 may have a profound effect on the epidemic of SARS-CoV-2 and may affect immune evasion by altering its interaction with IRF3 or downregulating its capability of MHC-I.

In the WT of SARS-CoV-2 ORF8 protein, the L84 residue is flanked by disulfides Cys83 and Pro85, which are highly conserved among ORF8 orthologs, indicating their indispensable roles for ORF8 (Flower et al., 2021). ORF8 was found to be involved in IFNß antagonism and MHC-I downregulation (Rashid et al., 2021; Zhang et al., 2021). However, the biological function of the residue 84 remains to be elucidated.

S24L is the second most frequent mutation in the SARS-CoV-2 ORF8 protein (Wang R. et al., 2020). S24L was found to increase the folding stability of the ORF8 protein and is associated with the dissemination of SARS-CoV-2 (Hassan et al., 2021; Wang R. et al., 2020). Therefore, it was assumed that this mutation may favor the function of ORF8 in immune evasion. HADDOCK docking analysis indicates its stronger binding with IRF3 (Figure 2B), which, in turn, may antagonize IFNß more efficiently.

The W45L substitution in ORF8 was first reported in Saudi Arabia and then circulated in Pakistan to cause more severe COVID-19 disease (Hassan et al., 2021). The circulation of this mutant in different countries indicated that W45L mutation may affect the function of the ORF8 protein through increasing its binding to IRF3 and enhancing its function in antagonizing IFNß. Our results support the above assumption. The HADDOCK docking score for W45L (IRF3-ORF8) was significantly bigger than that for ORF8 WT (Figure 2C), while the free binding energy for W45L was the highest among the ORF8 WT and mutants analyzed (Table 1). Collectively, the HADDOCK scores, the bonding network, the RMSD, the *Rg* scores, and the binding free energies enhanced the binding of W45L with IRF3 compared to ORF8 WT and other mutants. Moreover, the interaction interface with six hydrogen bonds and two salt bridges makes this substitution more likely to bind with IRF3 (Figure 2C). The stability analysis revealed by RMSD showed that the system is most stable for the W45L mutant (Figure 4A). Finally, hydrogen bonding (Figure 5B) and free energy binding (Table 1) affect the confirmation and structure of W45L that favors its strong binding with IRF3. Moreover, it was observed that over the course of mutations in ORF8, the function of ORF8 is going to be enhanced (Table 1).

In contrast, both the HADDOCK docking score and the binding free energy for L84S (IRF3-L84S) were close to the IRF3–ORF8 WT complex (Table 1), suggesting that this substitution may not enhance the binding of ORF8 with IRF3. Even the double-mutant V62L/L84S did not significantly affect its binding with IRF3. Our results are in line with the previous studies in which L84S substitution did not enhance the IFNß antagonism and the downregulation of MHC-I compared to ORF8 WT (Rashid et al., 2021; Zhang et al., 2021). We have found that the nuclear translocations of IRF3 by either overexpressing ORF8 WT or L84S in HEK293T cells were the same (Rashid et al., 2021). In another study, the overexpression of ORF8 WT in HEK293T cells or infected lung epithelial cells of hACE2 transgene mice significantly downregulated MHC-I. When overexpressing using L84S, similar effects were observed in the downregulation of MHC-I. Furthermore, the knockdown of ORF8 restored MHC-I expression (Zhang et al., 2021).

The current study was conducted to explore the interaction of the SARS-CoV-2 ORF8 protein and its mutants with the host IRF3 by using structural and biophysical analysis approaches to reveal the difference of binding energy and affinity. Our results indicate that this system is simple and useful to evaluate various ORF8 mutants with respect to its binding to IRF3 that may help in the evasion of the host immune system This investigation revealed the difference of ORF8 mutants in escaping the immune system compared to the ORF8 WT. However, experimental studies on various mutants of ORF8 are required to confirm their potential role in immune evasion by binding to IRF3.

We believe that SARS-CoV-2 may hijack other host proteins to enforce its infection and disease severity. It has been found that about 3.5% of COVID-19 patients had known autosomal-recessive (AR) deficiencies [interferon regulatory factor 7 (IRF7) and IFNAR1] and autosomal-dominant deficiencies (AD) [toll-like receptor 3 (TLR3), UNC93B1, TICAM1, TBK1, IRF3, IRF7, IFNAR1, and IFNAR2]. These findings suggested important roles of not only IRF3 or IRF7 but also TLR3 in the control of SARS-CoV-2 infection (Zhang et al., 2020a). This study further emphasizes the roles IRF7 along with IRF3 and further demanded biochemical and experimental approaches for rapid therapeutics of SARS-CoV-2 infections.

## Data Availability Statement

The datasets presented in this study can be found in online repositories. The names of the repository/repositories and accession number(s) can be found in the article/supplementary material.

## Author Contributions

FR: conceptualization, methodology, and writing – original draft preparation. MS and AS: bioinformatics analysis. ED, HW, and SC: writing – reviewing and editing. ST: supervision, conceptualization, and writing – reviewing and editing. All authors contributed to the article and approved the submitted version.

## Conflict of Interest

The authors declare that the research was conducted in the absence of any commercial or financial relationships that could be construed as a potential conflict of interest.
